# The functional neuroanatomy of face perception: from brain measurements to deep neural networks

**DOI:** 10.1098/rsfs.2018.0013

**Published:** 2018-06-15

**Authors:** Kalanit Grill-Spector, Kevin S. Weiner, Jesse Gomez, Anthony Stigliani, Vaidehi S. Natu

**Affiliations:** 1 Department of Psychology, School of Medicine, Stanford University, Stanford, CA 94305, USA; 2 Stanford Neurosciences Institute, School of Medicine, Stanford University, Stanford, CA 94305, USA; 3 Stanford Neurosciences Program, School of Medicine, Stanford University, Stanford, CA 94305, USA; 4 Department of Psychology, University of California Berkeley, Berkeley, CA 94720, USA; 5 Helen Wills Neuroscience Institute, University of California Berkeley, Berkeley, CA 94720, USA

**Keywords:** fMRI, human ventral visual stream, population receptive field

## Abstract

A central goal in neuroscience is to understand how processing within the ventral visual stream enables rapid and robust perception and recognition. Recent neuroscientific discoveries have significantly advanced understanding of the function, structure and computations along the ventral visual stream that serve as the infrastructure supporting this behaviour. In parallel, significant advances in computational models, such as hierarchical deep neural networks (DNNs), have brought machine performance to a level that is commensurate with human performance. Here, we propose a new framework using the ventral face network as a model system to illustrate how increasing the neural accuracy of present DNNs may allow researchers to test the computational benefits of the functional architecture of the human brain. Thus, the review (i) considers specific neural implementational features of the ventral face network, (ii) describes similarities and differences between the functional architecture of the brain and DNNs, and (iii) provides a hypothesis for the computational value of implementational features within the brain that may improve DNN performance. Importantly, this new framework promotes the incorporation of neuroscientific findings into DNNs in order to test the computational benefits of fundamental organizational features of the visual system.

## 1. Introduction

A central goal in cognitive and computational neuroscience is to understand how processing within the ventral visual stream enables rapid and robust recognition and classification of the visual input. Visual recognition is thought to be mediated by a series of serial computations that form a processing stream referred to as the ventral visual processing stream [1,2]. The ventral visual processing stream emerges in V1—the first cortical visual area that resides in the calcarine sulcus [3]—through a series of occipital visual areas, and ends in high-level visual regions in ventral temporal cortex (VTC), whose activation predicts visual perception and recognition [4–8].

**Figure 1.**
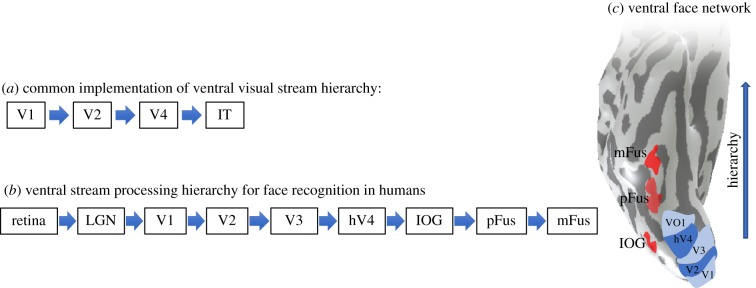
Ventral stream processing hierarchy for face recognition in humans. (*a*) A common ventral visual stream hierarchy based on the macaque visual system, implemented or referred to in the DNN literature. This hierarchy is adapted from [18], though some models begin in the retina [13]. (*b*) The ventral stream visual hierarchy of the human ventral face network. In the manuscript, we will only describe cortical regions starting from V1. This is a tentative suggestion based on present understanding of visual areas in the human brain (see 1*c*), but could be refined in future research when new knowledge (such as understanding the full connectivity pattern including feedback connections and bypass routes) will update this schematic. (*c*) Visualization of the ventral face network on an inflated cortical surface of an example participant showing the ventral aspect of occipito-temporal cortex (sulci in dark grey, gyri in light grey). Retinotopic areas are shown in shades of blue and labelled V1 to VO1. Face-selective regions are shown in shades of red and include IOG-faces (on the inferior occipital gyrus), pFus-faces (on the posterior fusiform gyrus) and mFus-faces (on the mid-fusiform gyrus). (Online version in colour.)

Recent neuroscientific discoveries have significantly advanced understanding of the function, structure and computations along the ventral stream processing hierarchy, revealing rich detail about their anatomical implementation, representations and computations (see reviews [9–13]). By anatomical implementation, we mean the physical features of the cortical tissue that act as the substrates performing the computation that produces accurate behaviour. Two important insights have emerged from neuroscience research: (i) the functional organization of the ventral visual stream is structured and (ii) it is reliable across individuals. That is, functional regions are consistently organized with respect to the cortical folding not only in V1 [3], but across the ventral stream more generally [14–17]. For example, the locations of retinotopic areas that contain maps of the visual field (V1-VO1, figure 1*a–c*) and face-selective regions (IOG-faces, pFus-faces, mFus-faces, figure 1*c*) are consistently arranged relative to the cortical folding and relative to each other [16,19,20]. These types of findings have led researchers to ask new questions such as (i) how do structural factors such as the underlying microarchitecture and white matter connections constrain the functional organization of the ventral stream? (ii) What is the computational purpose of this functional neural architecture?

In parallel, significant advances in computational models including hierarchical deep neural networks (DNNs) and technological advances that enable training DNNs using large and labelled image sets [21] have brought machine performance in recognition and classification of visual images to a level that rivals human performance [18,22–24]. This computational work has led to two important insights: (i) neurally inspired architectures trained with millions of images can produce optimal, human-like performance [22,23] and (ii) DNNs that learn by optimizing a behaviourally relevant cost function—such as categorization—better predict neural responses and representations in the primate and human brain, respectively, compared to other DNNs [18,25,26].

Because of these exciting recent advancements, this is an excellent time for the field of computational neuroscience to leverage advances in DNNs and to use them as a tool to probe the human visual system [27]. This will allow for a more mechanistic understanding of particular computations at different stages of the processing hierarchy and will provide crucial insights to the computational benefits of specific neural implementational features. Furthermore, perturbing aspects of the computational architecture will enable probing the necessity and sufficiency of specific neural implementational features for particular behaviours. Together, this can lead not only to foundational knowledge, but also to new approaches that could build predictions from computational models that may help rectify deficiencies and maldevelopment of the visual system.

To achieve these important goals, it is necessary for the field to implement and test neurally accurate computational models of the human visual system rather than models that are loosely ‘neurally inspired’. Therefore, the goal of this review is to use a model system within the ventral steam—the ventral face network—to illustrate how this goal can be achieved. We chose to focus on the ventral face network for several reasons: (i) it is a well-understood and studied system in both human [10,11,28–45] and non-human primates [46–56], (ii) functional regions in VTC which are causally involved in face recognition can be identified within each individual using functional magnetic resonance imaging (fMRI) [19,20,28,30], and (iii) the output computation of this system can be well defined in several levels of specificity ranging from categorizing a stimulus as a face to identifying a particular person (e.g. ‘this is Angela Merkel’). Thus, this review begins with a brief overview of the face recognition system in the human brain. The rest of the review is arranged in sections that describe specific neural implementational features of the ventral face network. For each feature, we consider similarities and differences between the functional architecture of the brain and DNNs, as well as provide a hypothesis for the computational value of this feature.

## 2. The ventral face network

To identify face-selective regions in the brain, participants are scanned in an fMRI scanner as they view faces and a variety of other stimuli such as body parts, objects, places and printed characters. In each subject, voxels in the ventral aspects of occipital and temporal cortex that respond significantly more strongly to faces than other stimuli are identified as face-selective. As shown in an example subject's inflated cortical surface (figure 1*c*), there are three face-selective clusters in the ventral visual stream, found bilaterally. One cluster is located in the inferior occipital gyrus (IOG) and is called IOG-faces (also referred to as the occipital face area [57]). A second cluster is located on the posterior-lateral aspect of the fusiform gyrus and is called pFus-faces [19]. A third patch is located on the lateral fusiform gyrus, about 1–1.5 cm anterior to pFus-faces, and tends to overlap the anterior tip of the mid-fusiform sulcus (MFS). This patch is referred to as mFus-faces [19]. In fact, in the right hemisphere, a 1 cm disc aligned with the anterior tip of the right MFS identifies approximately 80% of the face-selective voxels in the right mFus-faces [16]. pFus-faces and mFus-faces are often lumped together and referred to as the fusiform face area (FFA [28]). A characteristic of these ventral face-selective regions is that they respond to faces significantly more strongly compared to other stimuli [28,30], and this preference for faces is maintained across formats [29,58–61]. That is, both photographs and line drawings of faces evoke higher responses than photographs and line drawings of common objects. Selectivity to faces is also maintained when low-level features of the visual input are matched across faces and control stimuli (e.g. face silhouettes generate higher responses than shape silhouettes that are matched in contrast and area).

Ventral face-selective regions are thought to receive inputs from earlier retinotopic areas V1, V2, V3 and hV4 [62–64]. These earlier areas are labelled by their order in the visual processing hierarchy [62]. Each of these visual areas contains a map of the visual field (where the left hemifield is represented in the right hemisphere and vice versa). Retinotopic visual areas are thought to be connected to each other and also to the ventral face regions via axons [62,63]. Long-range axonal connections tend to be myelinated and form white matter tracts. Thus, some of the inputs from earlier visual areas to face-selective regions include portions of the inferior longitudinal fasciculus [65–67] (a large tract that connects the occipital lobe to the inferior aspect of the temporal lobe [68]). Additionally, ventral face-selective regions also have white matter connections to visual regions in the parietal cortex through vertical fasciculi such as the vertical occipital fasciculus (VOF [69–71]) and posterior arcuate fasciculus [70]. These vertical connections are thought to facilitate top-down modulations from the parietal-attention network to ventral regions [72]. However, in this review, we will concentrate on the feed-forward connections of the ventral face network.

Understanding this organization is useful for generating a tentative schematic of the processing hierarchy of the ventral face network (figure 1*b*). However, this is not often how the ventral stream processing hierarchy is portrayed in ‘neurally inspired’ DNNs. A typical DNN of the ventral stream based on the macaque visual system (shown in figure 1*a*) is portrayed as a feed-forward architecture progressing from V1 to V2 to V4 to IT (IT, or infero-temporal in the macaque, is thought to be homologous to human VTC). However, there are two main differences between the commonly implemented DNN and the human ventral stream. First, V3 is missing. This omission may be due to the fact that in macaque, V3 is substantially smaller than either V2 or V4 and there are direct white matter connections from V2 to V4. However, in the human brain, V3 is both equivalent in size to V2 [73,74] and larger than hV4 (figure 1*c*). Second, IT is often represented in DNN schematics as a single area. In the macaque, IT contains multiple subdivisions [55,75–80], and in humans, VTC is divided into several cytoarchitectonic areas [16,81–84], which contain more than 10 visual regions including: (i) two face-selective regions, pFus and mFus, figure 1*c*, (ii) additional domain-specific regions selective for places [85,86], bodies [87,88], objects [89] and characters/words [90], and (iii) several retinotopic areas: VO1/2 [91]; PHC1/2 [92]. Thus, we propose that the first step in building a neurally accurate feed-forward DNN for the human face recognition system is to include all the relevant areas in the human brain. Consistent with this idea, in the present manuscript, we will consider the following ventral face network: V1 → V2 → V3 → hV4 → IOG → pFus → mFus (figure 1*b*).

Why are we focusing only on the feed-forward aspect of this network? There are several reasons. First, humans can classify a stimulus as a face in less than 100 ms and recognize the identity of the face in approximately 150 ms [93,94]. This fast processing has prompted researches to suggest that face recognition does not necessitate top-down information and can be accomplished with fast, feed-forward processing. Second, face-selective responses in the fusiform gyrus emerge within 100–170 ms [38,95–98]. Third, as standard DNNs have a feed-forward architecture, we first compare them to the feed-forward components of the human visual system. Once these are well-understood, subsequent analyses will elucidate the role of non-hierarchical connections including the modulatory role of top-down connections from the parietal lobe [69,70,72] to the ventral stream, as well as the role of bypass connections [64].

As illustrated in table 1*a,b* and figure 1, there are some commonalities in the basic neural implementation of the ventral face network and DNNs. Critically, both types of networks enable hierarchical and feed-forward processing, which are thought to support two important computational benefits. First, the universal approximation theorem [99] has shown that these types of architectures can approximate any complex continuous function relating the input (here, the visual input) to the output (here, face recognition). Second, feed-forward processing with simple linear–nonlinear operations (which we will elaborate below) allows fast computations and, consequently, rapid performance (in our case, face recognition). Now that we have a foundation regarding the architecture of the ventral face network, we next turn to the computations that this structure produces.

**Table 1.** Comparison between several major characteristics of human ventral face network and deep neural networks.

property	human brain	deep neural network	hypothesized utility
*a.* hierarchical processing	**√**	**√**	enables computing of complex functions
*b.* feed-forward processing	**√**	**√**	speed
*c.* local computations	**√**	**√**	parallel processing
*d.* pRF/filter size increases along hierarchy	**√**	**√**	extraction of useful features
*e.* pRF/filter size increases with eccentricity	**√**	✗	solution to limited brain size
*f.* adjustable pRFs/filters	**√**	✗	task-optimized processing
*g.* learned pRFs/filters	**√**	**√**	flexibility; optimization for task and natural statistics
*h.* spatio-temporal pRFs/filters	**√**	✗	capture dynamics of natural environment

## 3. Basic computational unit in the visual system: receptive fields

In the human visual system, the basic computation is performed by receptive fields. A receptive field (RF) is the region in visual space that is processed by a neuron. Since neurons with similar RFs are spatially clustered, with fMRI we can measure the population receptive field (pRF)*—*the region in the visual field that is processed by the population of neurons in a voxel. RFs are often modelled by spatial filters that have linear–nonlinear operations. Example receptive fields that have been used to model responses in the visual system include Gaussians, difference of Gaussians and Gabor filter banks. These filtering operations are often followed by a nonlinearity such as a normalization, rectification or a compressive exponential nonlinearity [100–102].

These types of RF models have inspired the implementation of filters within DNNs. Indeed, each layer of a DNN contains a series of linear filter banks. Filters in each layer are applied uniformly on the input (image or output of prior layer) using a convolution operation. The output of the convolution can be followed by several mathematical operations to mimic neural responses: a thresholding nonlinearity (e.g. rectification or sigmoid), then pooling and, finally, normalization. Thus, filters in DNNs perform local operations on the image akin to those of receptive field models. The computations by pRFs/filters enable local, parallel processing of the image, which, in turn, increases computational efficiency (table 1*c*).

PRFs in the human brain have four fundamental characteristics that are interesting to consider when comparing to filters in DNNs. First, pRFs in the right hemisphere are centred in the left visual field, and those in the left hemisphere are centred in the right visual field. This is referred to as processing of the *contralateral* visual field. In other words, to increase parallel processing, the brain splits the visual input into two halves, each processed in a different hemisphere. DNNs typically process the entire image, though some implementations split processing across more than one graphics processing unit [22].

**Figure 2.**
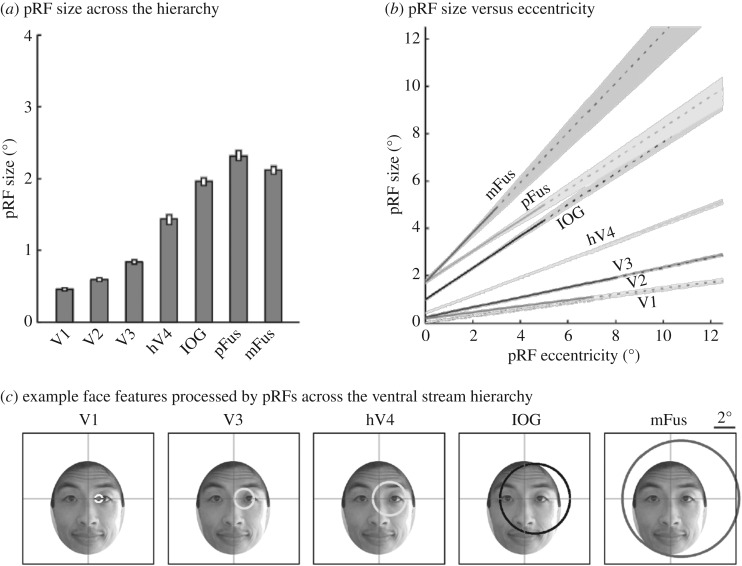
pRF properties across the ventral face network hierarchy. (*a*) Mean pRF size measured across the central 7° of each visual area. (*b*) There is a linear relationship between pRF size and pRF eccentricity across the ventral face network hierarchy. The slopes of lines relating pRF size and eccentricity increase across the processing hierarchy. (*c*) Example pRFs from the ventral face network. In each region, we illustrate a pRF centred at a 2° eccentricity on a face that is at typical viewing distance (approx. 1 m). The crosshair indicates the fixation point. Figure is adapted from [34].

Second, mean pRF size increases across the hierarchy of the ventral face network (figure 2*a*). The smallest pRFs are in V1 and the largest pRFs are in face-selective regions. For example, pRFs in face-selective regions are on average four times larger than those in V1 (figure 2*a*). This characteristic is also present in DNNs due to both the pooling operation and the repeated use of local convolutional filters. This results in a systematic increase in the extent of the visual image processed by filters as one ascends stages of the DNN. This increase in pRF/filter size is hypothesized to allow neurons/filters in higher stages to process information across several features, and perhaps even the entire object, rather than just local features as is the case for processing in lower stages of the network.

To give the reader an intuition of how mean pRF sizes in the ventral face network (figure 2*a*) relate to a real-life example, let us consider an example in which a face is viewed from a typical viewing distance (approx. 1 m away) and determine what facial features are processed by pRFs in different visual regions of the ventral face network. In this example, illustrated in figure 2*c*, a V1 pRF processes only the corner of the eye, a hV4 pRF processes the eye and the top of the nose, and a mFus-faces pRF processes the entire face. This example shows that the increase in pRF/filter size across the ventral visual hierarchy allows higher stages of the hierarchy to process more useful features for recognition (table 1*d*).

Third, in both the human and non-human primate visual system, RF size and consequently pRF size, increase with eccentricity [102–104] (figure 2*c*). That is, starting from the retina, and continuing throughout the entire processing hierarchy, RF size is not constant in a given region. Rather, both RFs and pRFs are smallest near fixation (centre of gaze) and increase roughly linearly with eccentricity (figure 2*b*). By contrast, filter size in DNNs is constant across each layer of the network. One reason why pRF size scales with eccentricity in the human and primate brain, but not in DNNs, may be limited resources. That is, the brain may need to optimize visual resolution given limited physical space as well as limited metabolic resources. The brain's solution to these limitations is to provide more resolution (smaller RFs) at the centre of gaze at the expense of less resolution (larger RFs) in the periphery (table 1*e*).

**Figure 3.**
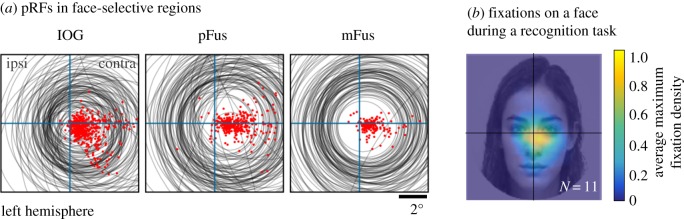
pRF properties in face-selective regions may affect the way people look at and fixate on faces. (*a*) Tiling of the visual field by pRFs in face-selective regions. pRFs are indicated by the grey circles, and their centres by the red dots. Ascending from face-selective IOG, to pFus, to mFus, pRFs become larger and become more concentrated on the centre of gaze. Adapted from [36]. (*b*) Fixation density on an example face during a face recognition task. Data are averaged across 11 adults. Colourbar indicates average maximum fixation density. Adults tend to fixate on the centre of the face when performing face recognition tasks. This behaviour puts the combined visual field coverage of pRFs in face-selective regions on informative facial features. Adapted from [105].

Fourth, in the human brain, pRFs in face-selective regions have a foveal bias. In face-selective regions, like in earlier visual areas, pRF centres are in the contralateral visual field (e.g. pRFs in the left hemisphere are centred in the right visual field, figure 3*a*). However, in face-selective regions, almost all of these pRFs overlap the fovea (figure 3*a*). We refer to this phenomenon as foveal bias. Given that pRFs in face-selective regions are large and overlap the fovea, this enables them to process information across both visual fields. Additionally, as one ascends from face-selective IOG, to pFus, to mFus, the foveal bias increases as pRF centres become more concentrated around fixation. Consequently, in face-selective regions, the centre of the visual field is more densely covered by pRFs than the periphery of the visual field [36,106–108].

It is appealing to hypothesize how this tiling of the visual field by pRFs in face-selective regions may relate to behaviour. One interesting behaviour is how people look at faces. A large literature indicates that during recognition, people tend to fixate on the centre of the face [109–113], as shown for the example in figure 3*b* (but see [114,115]). This fixation behaviour places pRFs in face-selective regions on the part of the face that has the most informative features for recognition [116–118]—that is, the eyes and nose.

## 4. PRFs in face-selective regions are modulated by the task

One interesting question is whether pRFs in the visual system are fixed or are modulated by task and behavioural goals. Several results show that attention and task may modulate pRF properties and this modulation seems to increase across the visual processing hierarchy [36,119,120]. Namely, attention has a more profound effect on pRFs in higher levels than lower levels of the hierarchy.

In our experiments, we tested if pRFs in the ventral face network are modulated by the task [36]. To do so, we measured pRFs by showing faces randomly in 25 locations while subjects centrally fixated on a stream of digits under two tasks: a digit task and a face task. In the digit task, participants indicated via a button press if two successively presented digits were the same, and in the face task, participants indicated if two successively presented images were of the same person.

Our results revealed three findings. First, attention to peripheral faces relative to central fixation increased pRF eccentricity in face-selective regions, but not early visual areas. That is, during the face task, pRFs in face-selective regions were further from fixation than during the digit task. In contrast, there were no changes to pRF eccentricity across tasks in early visual areas (V1–V3). Second, attention to faces increased pRF size in face-selective regions, but not early visual areas. In face-selective regions, pRF sizes were substantially larger during the face task than the digit task. For example, in mFus-faces, median pRF size increased from 1.8° in the digit task to 3.4° in the face task. Third, pRF gain in face-selective regions was larger in the face than digit task, but this was not apparent in early visual areas.

**Figure 4.**
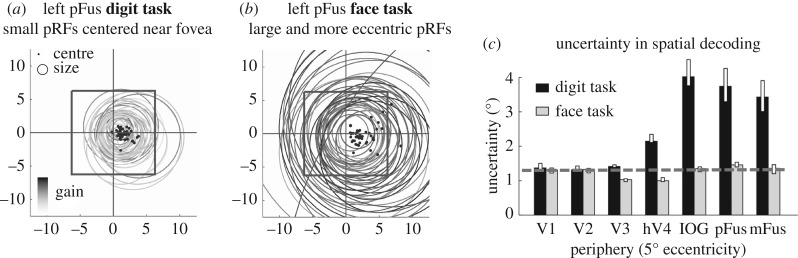
Attention to faces enhances representation and spatial precision in the periphery. (*a*) pRFs of left pFus-faces under the digit task, (*b*) pRFs of left pFus-faces under the face task. In *a* and *b*, pRFs are indicated by the circles, their centres are indicated by black dots, and their gain is indicated by the grey-level intensity (see colourbar). The black square indicates the size of a 5° image. (*c*) Spatial uncertainty in decoding the location of a face compared to an anchor face placed at 5° eccentricity based on the collection of pRFs in each task. Spatial uncertainty is lower during the face task (grey) than the digit task (black). Adapted from [36]. (Online version in colour.)

The combined effects of task on pRF size and eccentricity have a profound impact on the spatial representation of visual space by the collection of pRFs spanning each of the face-selective regions. This effect is illustrated in figure 4*a,b*: figure 4*a* illustrates the visual field coverage by pRFs of pFus-faces under the digit task, and figure 4*b* shows the pRFs of the same voxels during the face task. Notably, during the face task, pRFs are more scattered and extend further into the periphery than during the digit task. Thus, the consequence of attention to faces is enhanced representation of the periphery by pRFs of face-selective regions.

To quantify the effect of task on spatial acuity of the neural representation, we used a model-based decoding approach to quantify the spatial uncertainty obtained by pRFs measured under the different tasks. We found a significant four-fold reduction in spatial uncertainty in the periphery (5° eccentricity) in face-selective regions during the face task compared to the digit task (figure 4*c*). In contrast, spatial uncertainty obtained by pRFs in early visual areas remained stable across tasks. Interestingly, the spatial uncertainty obtained by pRFs in face-selective regions in the face task was no greater than that of V1 even though pRFs were substantially larger (figure 4*c*).

Thus, another difference between the human brain and DNNs is the finding of task-adjustable pRFs in higher stages of the hierarchy (table 1*f*). We speculate that this implementational feature allows the brain to adjust pRFs according to task demands and to enable more effective task-relevant processing. This task-based modulation is likely implemented in the brain via top-down connections. One candidate pathway that may facilitate such task-based modulation is the VOF. This white matter tract connects regions in the IPS that are involved in attentional gating with ventral stream regions, such as pFus-faces, thereby modulating responses in the ventral stream [72]. In addition to task-based modulations, experience and development also modify pRFs, which we address in the next section.

## 5. Both cortical and artificial networks are shaped by experience

One of the big contributions of the DNN literature for understanding biological visual systems is elucidating what types of filters are learned under different tasks. For example, in their seminal paper, Krizhevsky *et al.* [22] showed that training a DNN to categorize natural images generated V1-like oriented and colour-opponent filters in the first stage of their neural network. In other words, training the network to perform a categorization task using real images during training (ImageNet [21]) generated filters in the first convolutional layer that had similar properties to V1 receptive fields (RFs). Likewise, a large body of literature has examined the role of experience in shaping RF properties in V1 in species other than humans [121–124]. While the general retinotopic preference is present in infancy, likely due to wiring, experience is thought to be necessary to fine-tune RF properties of V1 neurons to obtain the adult-like specificity of their size, position and orientation tuning. This ability of DNNs and of the human brain to learn is key, as it gives the system considerable flexibility to learn the natural statistics of the visual world as well as to optimize the filters for extracting task-relevant properties (table 1*g*).

Presently, most DNNs use supervised learning (e.g. by labelling the category of training images) and algorithms such as back-propagation [125], which optimize a task-relevant cost function to learn relevant information. While humans may receive some supervised learning (e.g. a mother may name objects as they speak to their babies), it is thought that neurons in the brain can also fine tune their response properties via unsupervised learning from the natural statistics. Thus, a goal for computational modelling would be to develop a family of DNNs that learns from unsupervised training to better model biological visual systems.

**Figure 5.**
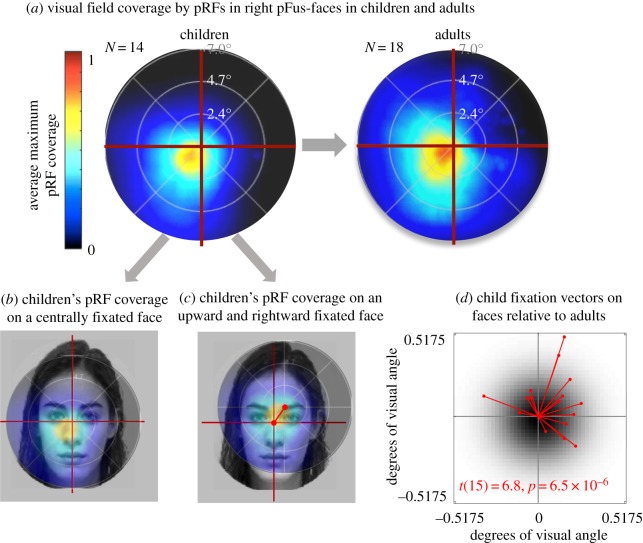
Development of visual field coverage in face-selective regions correlates with fixation patterns on faces. Adapted from [105]. (*a*) Visual field coverage by pRFs in right pFus-faces averaged across 14 children (left) and 18 adults (right). Colour indicates the average maximum pRF coverage in the central 7°. Crosshairs indicate fixation. (*b*) Placing the visual field coverage of right pFus-faces in children on the centre of the face would place pRF resources in a region without informative features. (*c*) Moving fixation upwards and rightwards (indicated by the red vector) places the visual field coverage of children's pRFs on the region of the face containing informative features. (*d*) Child fixation patterns on 16 faces compared to adults. Fixations are significantly shifted rightwards and upwards.

Notably, recent evidence suggests that the development of pRFs in higher visual areas, such as face-selective regions, continues well past infancy and during childhood [105] even as pRFs in V1 and other early visual areas are adult-like by age 5 [105,126,127]. In a recent study, we measured pRF properties and the visual field coverage of pRFs in face-selective regions of school-age children and adults [105]. We found substantial developmental changes in the visual field coverage in face-selective regions from childhood to adulthood. As illustrated in figure 5*a*, the right pFus-faces of children shows a foveal bias (higher density of the visual field coverage around the centre of gaze), and a coverage of the left, lower visual field. In adults, right pFus-faces also shows a foveal bias. However, compared to children, the visual field coverage in adults' right pFus-faces (i) expands to the upper and right (ipsilateral) visual field and (ii) the foveal bias increases. These data show that pRF properties in face-selective regions continue to develop after age 5.

What are the implications of the development of visual field coverage by pRFs? One prediction from our findings is that face viewing behaviour should differ across age groups. In other words, we predict that if pRFs in face-selective regions guide viewing behaviour, then the differing visual field coverage of pFus-faces across age groups would result in differing fixation patterns on faces across age groups. To illustrate this point, consider figure 5*b*, which shows the pRF coverage of children's right pFus-faces superimposed on an example face. Central fixation, as performed by a typical adult, will put the visual field coverage of the child's pFus-faces on the edge of the nose and cheeks, which do not contain useful information for face recognition. In other words, a child presented with the example face should not fixate on the centre of the face as it will place the visual field coverage of pFus-faces outside the region with useful features. Instead, the child should shift their fixation upwards and rightwards (figure 5*c*), as this fixation behaviour will place the visual field coverage of right pFus-faces on informative features for face recognition. It turns out that this is precisely what children do. Comparison of fixation patterns on faces in children and adults indicate that children's fixations on faces are indeed consistently shifted upwards and rightwards compared to adults (figure 5*c*), thus putting the pRFs of face-selective regions on the informative features. A second implication from our results is that fixation patterns on faces, as well as pRFs in face-selective regions, may be shaped by lifelong experience and consequently, may vary across cultures with different stereotypical viewing of faces (e.g. [115]. Future research comparing pRFs across cultures with distinct face viewing norms can address this question.

## 6. Neural sensitivity to face identify develops from childhood to adulthood

While development of pRFs in face-selective regions is related to face viewing patterns, this development does not explain why face recognition performance in adults is better than in children. We hypothesized that another facet of functional development may be increased neural sensitivity to face identity. Increased neural sensitivity may lead to increased perceptual sensitivity and consequently, better face recognition performance.

**Figure 6.**
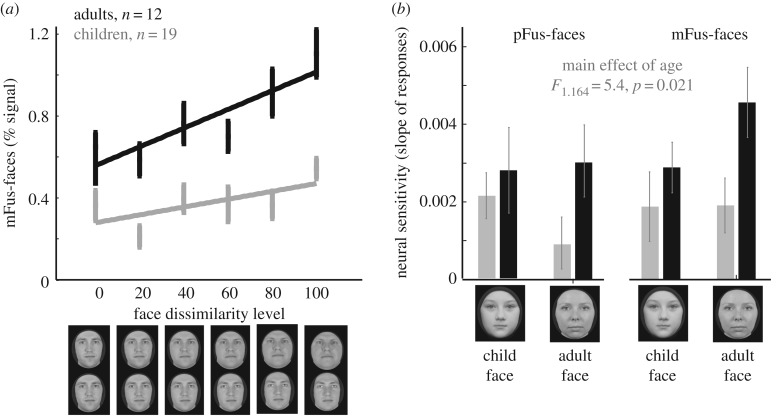
Sensitivity to face identity develops from childhood to adulthood. (*a*) Average response in mFus-faces across 12 adults (19–34 years old, black) and 19 children (5–12 years old, grey) to faces that vary in their level of dissimilarity. The slope of this line indicates sensitivity to face identity. The *x*-axis indicates the dissimilarity between faces in a trial starting from 0 (identical) to 100 (different real-world individuals) in increments of 20%. In order to systematically vary dissimilarity among faces, Natu *et al*. [128] morphed a target face to six different identities and varied the weighting of the source and target faces. In each 4-s trial, subjects viewed six faces from these morphs. In different trials, subjects viewed male and female faces as well as adult and child faces. (b) Slope of the line relating amplitude of response to face-dissimilarity in children (grey) and adults (black) as they viewed adult and child faces. Data in this figure are adapted from [128]; *Error bars*: standard error of the mean.

To test if neural sensitivity to face identity develops from childhood to adulthood, in a different study [128], we used a parametric fMRI-adaptation (fMRI-A [89,129]) experiment. In adults, responses to repetitions of the same face are lower than responses to different faces, due to neural adaptation [89,129]. Importantly, the level of fMRI-A is dependent on the level of face similarity [130–132]. That is, the more similar the faces are, the larger the fMRI-A. Therefore, we designed an experiment in which we systemically varied face similarity and tested if the slope of the function relating neural responses to face dissimilarity (defined as neural sensitivity) varies across age groups [128]. We predicted that if neural sensitivity to faces develops, the slope of this line will be steeper in adults than children. Indeed, that is precisely what we found. Interestingly, this development was specific to the face-selective regions of the ventral face network (figure 6*a*). Further analyses indicated that the neural sensitivity to face identity is also influenced by recent experience and the social salience of faces. In pFus-faces, children had higher neural sensitivity to child than adult faces, and in mFus-faces, adults had higher neural sensitivity to adult faces than child faces (figure 6*b*). Notably, the degree of neural sensitivity was correlated to perceptual discriminability of face identity. That is, subjects with higher neural sensitivity to faces in pFus- and mFus-faces had higher perceptual sensitivity. Together, these data show that both pRFs and the neural sensitivity to face identity develop from childhood to adulthood. Furthermore, this development was coupled with improved perceptual discriminability.

## 7. Receptive fields in the visual system process changes across both space and time

Finally, another key difference between processing by filters in the brain and filters in DNNs emulating the ventral stream is their temporal sensitivity. Typical DNNs for recognition, categorization and face identification contain temporally-static filters. In contrast, the visual system has dynamic RFs (table 1*h*). For example, electrophysiological recordings in macaque V1 have found that V1 RFs are best understood as spatio-temporal filters [133–137] in which RFs process changes in the visual input across both space and time.

**Figure 7.**
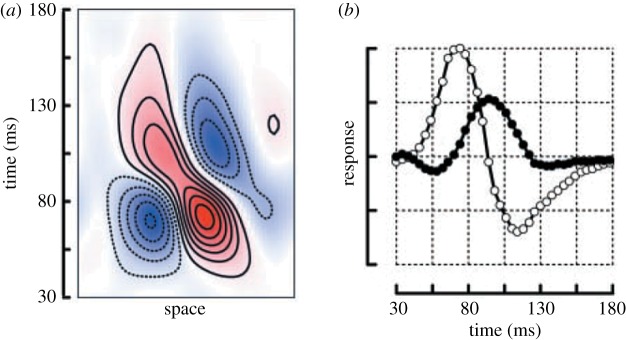
Example spatio-temporal receptive fields (RF) in macaque V1. (*a*) Example spatio-temporal receptive field recorded in macaque V1. This filter has both spatial (*x*-axis) and temporal (*y*-axis) tuning. (*b*) Example temporal characteristic of a monophasic (black) and biphasic (grey) temporal RF in macaque V1. Adapted from [138]. (Online version in colour.)

Electrophysiology studies commonly report two types of temporal filters in V1: monophasic and biphasic filters [138–140]. Monophasic temporal filters compute the ongoing sustained visual response—that is, they produce elevated firing when a visual stimulus is present. In contrast, biphasic temporal filters compute the temporal derivative of the visual input, indicating when there is a change in the visual stimulus. Thus, spatio-temporal filters compute time-varying aspects of the visual stimulus. For instance, in V1 they process changes in contrast and/or orientation over time (figure 7).

While initial research on spatio-temporal filters [133,137,138] was focused on understanding properties of neurons that code the direction of visual motion (which are found in V1 and MT), recent evidence suggests that such transient and sustained temporal channels are found not only in V1, but also across the visual system [101,141] including the ventral stream [141]. This finding is somewhat surprising because recognition can be done from brief, static images [93,94,142] and visual motion does not strongly modulate responses in ventral face-selective regions [143]. The combination of this recent evidence leads to the following intriguing question: What is the computational purpose of spatio-temporal filters in the ventral face network and the ventral visual stream more broadly?

We speculate that spatio-temporal filters may serve several computational goals. First, in contrast to artificial DNNs in which the visual input is introduced one image at a time, the visual input in the natural worlds is continuous, except for discontinuities introduced by eye movements. Therefore, spatio-temporal filters may parse the visual input. For example, biphasic temporal filters may be useful for detecting novel stimuli (e.g. a new face) and monophasic temporal filters may code sustained aspects of the visual input [141]. Second, spatio-temporal filters may compute correlations across space and time from the visual input that may function to bind incident two-dimensional views of the same object together [144,145] (e.g. linking among different face views belonging to the same individual), which is a process that may be particularly useful for unsupervised learning [145–147]. Third, some items in the world, such as bodies and animate beings, are non-rigid [148]. Thus, spatio-temporal filters may aid in computing dynamic features, which may be particularly useful for recognition of non-rigid stimuli. Therefore, a productive avenue for future DNN research would be to implement dynamic spatio-temporal filters within the DNN architecture to test these hypotheses and to determine the added value of dynamic compared to static filters.

## 8. Using deep neural networks to test the computational utility of implementational features of the neural architecture

Throughout this review, we described important implementational features of the human ventral face network, compared these features with present DNN architectures, and proposed hypotheses for the computational utilities of various implementational features. These ideas are summarized in table 1. We are hopeful that these neural features will be incorporated into modern DNNs to generate a new class of neurally accurate computational models of the ventral stream and specifically of the face network. To make DNNs neurally accurate, there is a need to implement neural features that are presently absent including: (i) filters that sample the visual field in a non-uniform manner, (ii) filters that can be adjusted to accommodate varying task demands, (iii) temporally dynamic filters, (iv) a correct number of processing stages, and (v) recurrent and top-down connections. Adding these features into DNNs may (i) enhance understanding of the computations along the ventral stream, (ii) likely improve the predicted brain responses to a variety of stimuli, and (iii) provide important insights to the hypothesized utility of various architectural features of the human brain. As the interplay between neuroscience and computer science increases, it is important to consider that comparisons between DNNs and the human brain can be done at many levels. For example, DNNs can be used to predict responses of single neurons or fMRI voxels. Alternatively, one can compare the types of representational spaces emerging in DNNs compared to the brain, or examine if the spatial layouts of these representations are similar to the spatial layouts across the cortical sheet [18,25,26]. We believe that each of these different comparison levels (as well as others that we have not considered) are useful, because they will provide important insights to cortical computations, as well as anatomical and functional constraints that serve as the infrastructure for these computations.

Critically, if these neurally accurate DNNs prove to be better models of brain responses as well as human behaviour compared to standard DNNs, we can use these computational models to test the role of specific implementational features on both brain responses and recognition behaviour. For example, we have shown that pRFs in face-selective regions have a foveal bias and that adults tend to fixate on the centre of the face during recognition. We hypothesized that this viewing behaviour places pRFs of face-selective regions on the informative features for recognition. This hypothesis can be tested by a neurally accurate DNN in which lower layers have filters that scale with eccentricity and higher layers have foveally biased filters. For example, using such a network trained on face recognition, we can test if better recognition occurs when an input image of a face is presented either (a) centrally, at the network's ‘fovea’ or (b) off-centre.

Another enigma that can be resolved with neurally accurate DNNs is why there are three face-selective regions in the ventral face network and what computational goal they may serve. To investigate this question, one can generate a family of DNNs in which the number of higher layers vary (even as lower layers are held constant). Using this framework, researchers could directly test what features emerge in higher layers, as well as how the number of layers may affect (i) performance, (ii) the efficiency of computations or (iii) the speed and accuracy of learning. Nonetheless, we acknowledge that this comparison will be complex, as there may not be a 1-to-1 correspondence between layers in a DNN to stages (or brain areas) spanning the ventral visual hierarchy.

In sum, neuroimaging research has advanced our understanding regarding the functional architecture of the human ventral face network. Importantly, incorporating these recent findings in up-to-date computational DNNs will further advance the field by providing enhanced understanding of the computational benefits of specific implementational features of the human brain.

## Data accessibility

Code related to figure 1 which contains the experiment used to define face-selective regions by contrasting responses to faces versus other images can be found here: https://github.com/VPNL/fLoc. Data and code related to figures 2–4 can be found here: http://kendrickkay.net/vtcdata/. Code related to figure 5 can be found here: https://github.com/VPNL/pRF_Development.
